# Immunohistochemical analysis reveals variations in proteasome tissue expression in *C*. *elegans*

**DOI:** 10.1371/journal.pone.0183403

**Published:** 2017-08-17

**Authors:** Elisa Mikkonen, Caj Haglund, Carina I. Holmberg

**Affiliations:** 1 Research Programs Unit, Translational Cancer Biology Program, University of Helsinki, Helsinki, Finland; 2 Department of Surgery, University of Helsinki and Helsinki University Hospital, Helsinki, Finland; East Carolina University, UNITED STATES

## Abstract

The ubiquitin-proteasome system (UPS) plays a crucial part in normal cell function by mediating intracellular protein clearance. We have previously shown that UPS-mediated protein degradation varies in a cell type-specific manner in *C*. *elegans*. Here, we use formalin-fixed, paraffin-embedded *C*. *elegans* sections to enable studies on endogenous proteasome tissue expression. We show that the proteasome immunoreactivity pattern differs between cell types and within subcellular compartments in adult wild-type (N2) *C*. *elegans*. Interestingly, widespread knockdown of proteasome subunits by RNAi results in tissue-specific changes in proteasome expression instead of a uniform response. In addition, long-lived *daf-2(e1370)* mutants with impaired insulin/IGF-1 signaling (IIS) display similar proteasome tissue expression as aged-matched wild-type animals. Our study emphasizes the importance of alternate approaches to the commonly used whole animal lysate-based methods to detect changes in proteasome expression occurring at the sub-cellular, cell or tissue resolution level in a multicellular organism.

## Introduction

Correct maintenance of the lifecycle of proteins, i.e., from their synthesis to their degradation, is critical for all organisms. In eukaryotic cells, intracellular protein turnover is mediated by two evolutionarily conserved systems: the autophagy-lysosome pathway and the ubiquitin-proteasome system (UPS). In the UPS, polyubiquitin-marked proteins are routed to a large protease complex, the 26S proteasome, for their proteolytic degradation [1]. The 26S proteasome is a multi-subunit protease complex consisting of a proteolytic core particle known as the 20S proteasome capped by one or two 19S regulatory particles [2]. Alternatively, the 20S can associate with the 11S complexes or PA200/Blm10 (proteasome activator 200 kDa/bleomycin resistance protein-10) [3]. The 20S proteasome is composed of four stacked heteroheptameric rings: two α- and two β-rings, each containing seven α- or β-subunits. The proteolytic activity of the proteasome is conveyed by the β-rings [4]. Proteasome genes are highly conserved and the *Caenorhabditis elegans (C*. *elegans)* orthologues of human 20S proteasome subunits are referred to as proteasome alpha subunits 1 to 7 (*pas-1-7*) and proteasome beta subunits 1 to 7 (*pbs-1-7*) [5].

Regulation of proteasome function is complex, affecting its abundance, composition and proteolytic processing capacity. Proteasome activity can be directly regulated through post-translational modifications of the proteasome, such as phosphorylation by protein kinase A [6]. The capacity for proteasome’s substrate degradation can also be affected by associated ubiquitin ligases (E3s) and deubiquitinating enzymes (DUBs) [7, 8]. Proteasome abundance is at least partly regulated through a negative feedback loop, also known as the “bounce-back” effect, by which transcription of proteasome subunits is promoted upon impaired cellular proteasome activity [9, 10]. In yeast, this effect is regulated by the transcription factor Rpn4p, which is also a proteasome subunit. In mammalian cells and in *C*. *elegans*, the Rpn4p-unrelated transcription factors Nrf1/2 and SKN-1 mediate this bounce-back effect, respectively [10–14]. Other transcription factors affecting proteasome abundance are FOXO (forkhead box O) transcription factors DAF-16 in *C*. *elegans* and FOXO4 in mammals by influencing transcription of the 19S regulatory particle subunit Rpn6 [15, 16]. However, regulation of basal proteasome gene expression is for the moment fairly unknown.

We have previously developed fluorescent-based reporters for *in vivo* measurement of UPS activity in live *C*. *elegans* and shown that UPS-mediated protein degradation varies in a cell-type and age-dependent manner [7, 17]. We have also demonstrated that long-lived *daf-2(e1370)* mutants with reduced insulin/IGF-1 (insulin-like growth factor) signaling (IIS) display increased *in vivo* UPS activity in body-wall muscle cells and intestinal cells, without any detectable difference in proteasome amount in whole animal lysates [7]. Although Western blotting and mass spectrometry-based quantitative proteomics on whole animal homogenates are currently the most commonly used methods to study protein abundance in *C*. *elegans*, these methods exclude tissue and cell-type specific protein expression analysis. To address this issue, we have performed immunohistochemistry on formalin-fixed, paraffin-embedded *C*. *elegans* to semi-quantitatively study proteasome expression at a tissue- and cell-level resolution. To date, immunohistochemistry in *C*. *elegans* has only been reported in a few studies on localization and accumulation of the aggregation-prone human tau and amyloid-beta proteins [18, 19]. Here, we utilize immunohistochemistry to detect expression of endogenous proteins in *C*. *elegans*.

Our study reveals that wild-type *C*. *elegans* displays variable proteasome immunoreactivity within different cell types and subcellular compartments. We also show that proteasome expression is affected in a tissue-specific manner upon widespread targeted 20S α-subunit knockdown. Furthermore, we demonstrate that impaired insulin/IGF-1 signaling of *daf-2(e1370)* mutants does not affect tissue expression of the proteasome, when compared to wild-type animals, supporting a role for tissue-specific post-translational regulation of UPS activity.

## Materials and methods

### Nematodes and growth conditions

*C*. *elegans* strains were grown under standard conditions at 20°C as previously described [20]. The following strains were obtained and used from the Caenorhabditis Genetics Center (CGC): N2 [wild-type Bristol isolate], and CB1370: *daf-2(e1370)III*. GR1702: *Is*[*sur-5*::*GFP*] strain was a kind gift from Dr. Gary Ruvkun and previously described [21].

### Gel electrophoresis followed by immunoblot analysis or in-gel proteasome activity assay

Age-synchronized N2 animals at day 1 of adulthood (4 days old) were collected in M9 buffer (22 mM KH_2_PO_4_, 41 mM Na_2_HPO_4_, 8,5 mM NaCl, and 19 mM NH_4_Cl) prior to freezing in -80°C. Animals were homogenized using a dounce homogenizer and native gel lysis buffer, as previously described [7]. Western blot samples were separated on a SDS- (sodium dodecyl sulfate) polyacrylamide gel and blotted onto a nitrocellulose membrane using Trans-Blot Turbo Transfer System (Bio-Rad). Proteasome 20S α-subunits antibody (MCP231, Enzo Life Sciences) in 1:1000 dilution and anti-α-tubulin antibody (T5168, Sigma) in 1:10000 dilution were used in blotting. The anti-20S antibody recognizes six out of the seven α-subunits of the 20S proteasome (α1, α2, α3, α5, α6 and α7). Due to similar molecular masses of these α-subunits only four separate bands are usually detected. Signal intensity of the immunoblots was adjusted and quantified using image processing software based on ImageJ (Fiji Life-line version, 2015 December 22). Native gel electrophoresis and the in-gel proteasome activity assay were performed as earlier reported with a few exceptions [7]. Gels were run in an ice bath for 30 minutes at 20 mA and then for 1 hour and 40 minutes at 40 mA. Following gel electrophoresis, the gel was either blotted onto a nitrocellulose membrane prior to immunoblotting with the anti-20S α-subunits antibody or incubated in developing buffer containing 160 μM of fluorogenic proteasome substrate suc-LLVY-AMC (succinyl-leu-leu-val-tyr-7-amino-4-methylcoumarin) (l-1395, Bachem). Developed gels were exposed to ultraviolet light and imaged with MultiImage Light Cabinet using FluorChem 8900 software (Alpha Innotech Corporation). Signal intensity was adjusted using Fiji Life-line version, 2015 December 22.

### Immunohistochemical analysis

Immunohistochemical analyses were performed on age-synchronized animals. Animals at 1 day of adulthood (4-day old N2, and 4-day old *sur-5*::*gfp* transgenic animals, and 5-day old *daf-2(e1370)* mutants) were washed in M9 buffer and fixed in 10% (v/v) phosphate buffered formalin for 20 minutes at room temperature. Formalin-fixed animals were then embedded in agar (2% w/v in Milli-Q), and fixed again in 10% (v/v) phosphate buffered formalin overnight, prior to processing the agar blocks into paraffin. Paraffin-embedded agar blocks were cut into 4-μm sections, fixed on slides and dried for 12 to 24 hours at 37°C. Sections were deparaffinized in xylene and rehydraded through gradually decreasing concentrations of ethanol to distilled water. For antigen retrieval slides were treated in a PreTreatment module (Lab Vision) in 0,05 M Tris-HCl buffer, pH 8,5 for 20 minutes at 98°C prior to immunohistochemistry. Immunohistochemical staining was performed using Dako REAL^TM^ EnVision^TM^ Detection System, Peroxidase/DAB+, Rabbit/Mouse kit (Dako) as reported earlier [22]. The slides were incubated for an hour at room temperature with either the proteasome 20S α-subunits antibody (MCP231, Enzo Life Sciences) in 1:2000 dilution or anti-GFP antibody (11814460001, Roche) in 1:5000 dilution. The immunoreaction was developed with a 3,3’-diaminobenzidine tetrahydrochloride (DAB) chromogen (DAKO) resulting in a brown reaction product. Samples were counterstained with Mayer’s haematoxylin solution, rinsed in tap water and dehydrated through gradually increasing concentrations of ethanol to xylene before being mounted with Pertex^®^ mounting medium (HistoLab). Antibody specificity in the section was demonstrated by pre-incubating the antibody with purified human 20S proteasome (E-360, R&D Systems) in 1:100 dilution for an hour at room temperature before incubated with the sections. The slides were imaged either with a Zeiss Axio Imager.Z1 upright epifluorescence microscope connected to an AxioCam Mrc5, 5 megapixel color CCD camera (Zeiss) and Zen 2 software (Zeiss) or a Zeiss Axio Imager.Z2 upright epifluorescence microscope connected to an AxioCam 105 color, 5 megapixel color CMOS camera (Zeiss) and Zen 2 pro software (Zeiss). Images were acquired either with 10x 0.3 NA, 40x 0.75 NA EC Plan Neofluar objectives, or with a 63x 1.4 NA Plan Apochromat objective. Micrographs were processed with Photoshop CS6 software (Adobe Systems). Evaluation of immunostaining was done independently by two investigators. Staining intensity was scored as 0 for negative, 1 for mild, 2 for moderate, and 3 for strong positive immunoreactivity.

### *C*. *elegans in vivo* imaging

Age-synchronized animals at day 1 of adulthood (4-day old animals) were mounted on 3% agarose pads and immobilized using 0,5 mM levamisole in M9. Animals were imaged with a Zeiss Axio Imager.Z2 upright epifluorescence microscope connected to an Orca Flash 4.0 LT, 4 megapixel monochrome sCMOS camera (Hamamatsu) and Zen 2 pro software (Zeiss). Images were acquired with 40x 0.75 NA EC Plan Neofluar objective. Micrographs were processed with Photoshop CS6 software (Adobe Systems).

### *C*. *elegans* RNA-interference (RNAi)

RNAi was performed using the feeding protocol as described earlier [23]. Bacterial strain HT115 carrying the empty p*L4440* expression vector was used as a control. To induce double-stranded RNA expression in the bacteria, 0,4 mM isopropyl β-D-1-thiogalactopyranoside (IPTG) (I6758, Sigma) was used, and its concentration was further increased to 0,8 mM before seeding the plates. To avoid developmental defects of proteasome downregulation, age-synchronized N2 animals were placed on control RNAi feeding plates as L1 larvae (day 1) and transferred as L3 larvae (day 2) to RNAi feeding plates targeting *pas-5* or *pas-6* alone (F25H2.9 and CD4.6, respectively, J. Ahringer RNAi library), or targeting both *pas-5* and *skn-1* (T19E7.2, Vidal ORFeome-Based RNAi library). Combined RNAi of *pas-5* and *skn-1* was performed by mixing in a 1:1 volume ratio (taken into account the optical density) *pas-5* and *skn-1* RNAi bacterial cultures prior seeding. Accordingly, in this experimental setup the RNAi bacteria targeting *pas-5* or *skn-1* alone were diluted in a 1:1 volume ratio with pL4440 bacteria. To knockdown *gfp*, age-synchronized *sur-5*::*gfp* animals were exposed to *gfp* RNAi (the bacterial clone was a gift from Dr. Gary Ruvkun) as L1 larvae (day 1). Animals were harvested at day 1 of adulthood (day 4) for analyses.

### Statistical analysis

Statistical significances were determined by Student’s t-test (two-tailed, paired).

## Results

### Proteasome expression varies in different tissues in adult wild-type *C*. *elegans*

**Fig 1:**
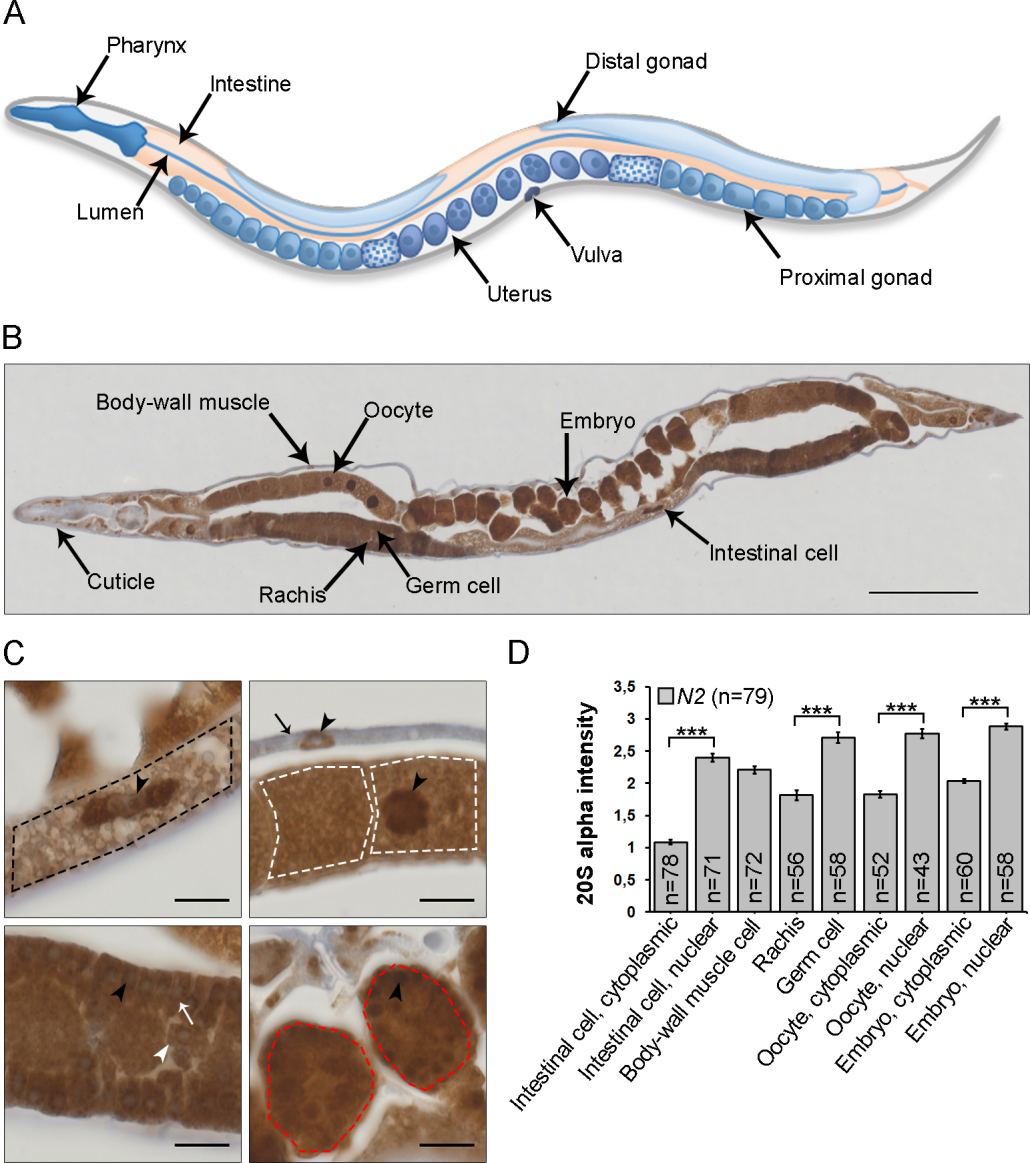
Proteasome tissue expression in wild-type (N2) *C*. *elegans* sections. (A) Schematic picture of the body plan and tissues of an adult hermaphrodite *C*. *elegans*. (B) Proteasome immunostaining in a longitudinal whole animal section (4μm) of 1-day old adult *C*. *elegans*. Specific cell types, rachis, and extracellular cuticle are indicated by arrows. Scale bar: 100 μm. (C) Large magnification images of different areas of the same *C*. *elegans* as presented in (B) showing proteasome immunoreactivity in an intestinal cell (upper left panel, outlined with a black dash line), body-wall muscle cell (upper right panel, indicated by a black arrow), oocytes (upper right panel, outlined with white dash lines), germ cells (lower left panel, indicated by a white arrow), rachis (lower left panel, indicated by a white arrowhead), and embryos (lower right panel, outlined with red dash lines). Black arrowhead points to the nucleus. Scale bars: 10 μm. (D) Quantification of immunoreactivity. Graph shows the mean staining intensity of three independent experiments (n = number of animals). Error bars: ± SEM. ***p < 0,001.

To study proteasome tissue expression in adult wild-type (N2) *C*. *elegans*, we performed immunohistochemistry on sections of formalin-fixed, paraffin-embedded animals. To detect the proteasome, we used an anti-20S antibody, which has previously been shown to recognize proteasome α-subunits resolved on SDS-PAGE (S1A Fig and [7]). Analysis of whole animal lysates by immunoblotting under non-denaturing conditions demonstrated that this antibody recognizes native α-subunits integrated in proteolytically active proteasome complexes (S1B and S1C Fig). Importantly, no bands corresponding to free α-subunits were detected, even after longer exposures, demonstrating that the amount of free α-subunits is non-existing or very low in the lysates (S1B Fig, data not shown). Immunostaining of the proteasome in adult *C*. *elegans* sections showed clear positive immunoreactivity in most tissues and cell types (Fig 1A and 1B). The immunoreactivity was strongly reduced when the primary antibody was omitted or pre-absorbed with purified human 20S proteasome, validating the specificity of the proteasome immunostaining in the sections (S1D Fig). We detected proteasome expression in most somatic cell types, such as intestinal cells, body-wall muscle cells and in the gonad, including germ cells, oocytes and embryos, but not in the extracellular cuticle (Fig 1B and 1C). In all cell types, immunoreactivity was weaker in the cytoplasm compared to the nucleus and/or perinuclear space (Fig 1C and 1D). This effect was most prominent in intestinal cells (Fig 1C and 1D). To validate suitability of enzymatic peroxidase-based immunohistochemistry for comparable antigen detection in different tissues and sub-cellular compartments, we used a transgenic strain expression GFP as a fusion protein from the ubiquitous *sur-5* promoter and compared the GFP immunoreactivity in sections to the GFP fluorescence in live animals. Positive GFP immunoreactivity was detected in the same tissues, cells and sub-cellular compartments as the GFP fluorescence signal and the intensities of the immunoreactivity correlated with the GFP fluorescence intensities (S2A and S2B Fig). Taken together, our data show that proteasome immunoreactivity varies in different cell types and within cellular compartments in 1-day old adult wild-type *C*. *elegans*.

### Targeted proteasome subunit knockdown leads to tissue-specific responses in proteasome expression

**Fig 2:**
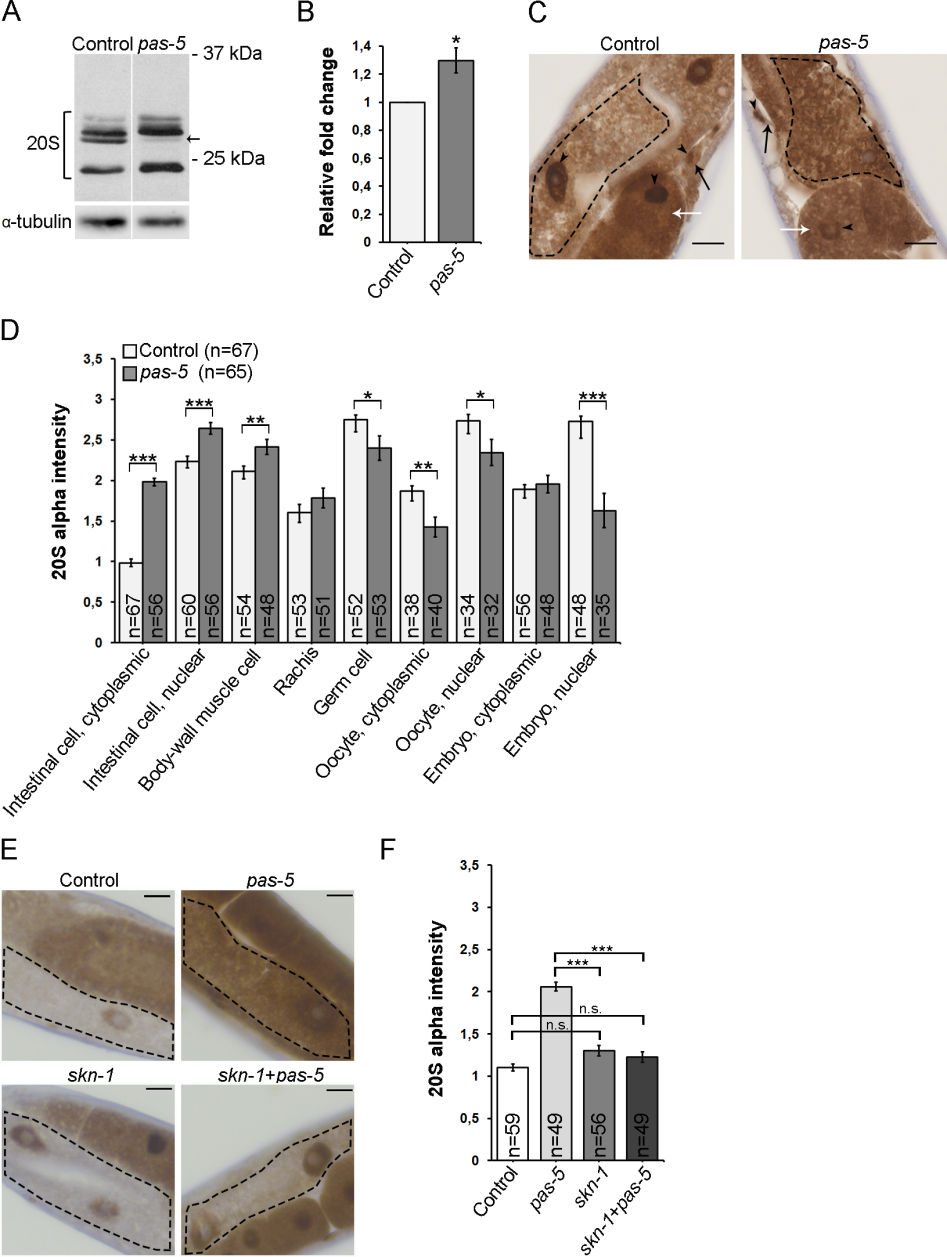
Targeted *pas-5* RNAi results in SKN-1–dependent intestinal upregulation of proteasome α-subunits. (A) Lysates of wild-type animals treated with control or *pas-5* RNAi separated on SDS-PAGE prior to immunoblotting with anti-20S α-subunits antibody (upper panels). Black arrow points to a band expected to correspond to proteasome α-subunit 5. Lower panels show α-tubulin expression. The samples were run on the same gel. (B) Quantification of the immunoblots corresponding to the total intensity of the displayed bands. Graph shows mean relative fold change in levels of 20S α-subunits, when normalized against α-tubulin. Error bars ± SEM of three independent experiments. *p < 0.05. (C) Images of an adult wild-type animal fed control (left panel) or *pas-5* RNAi bacteria (right panel) presenting proteasome immunoreactivity in intestinal cells (outlined with black dash lines), oocytes (indicated by white arrows), and body-wall muscle cells (indicated by black arrows). Black arrowhead points to nucleus. Scale bars: 10 µm. (D) Quantification of immunoreactivity. Graph shows the mean staining intensity of three independent experiments (n = number of animals). Error bars ± SEM. ***p < 0,001, **p < 0,01, *p < 0,05. (E) Images presenting proteasome immunoreactivity in the cytoplasm of intestinal cells (outlined with black dash lines) of wild-type animal fed with control (upper left panel), diluted *pas-5* (upper right panel), diluted *skn-1* RNAi bacteria (lower left panel) or a culture containing both *pas-5* and *skn-1* RNAi bacteria in 1:1 volume ratio (lower right panel). Scale bars: 10 μm. (F) Quantification of cytoplasmic proteasome immunoreactivity in the intestinal cells. Graph shows the mean staining intensity of three independent experiments (n = number of animals). Error bars ± SEM, ***p < 0,001, n.s. = non-significant.

We have previously shown that knockdown of the proteasome subunits *pas-5* or *rpn-2* by RNAi result in dramatic upregulation of the transcriptional reporters of the proteasomal subunits *pbs-4*, *rpt-5* and *rpn-11*, as well as of the RPN-11::GFP translational fusion protein in *C*. *elegans* [14]. This so-called “bounce-back” response, *i*.*e*. a compensatory upregulation of proteasome subunit gene expression upon inhibition of the proteasome, was first described in mammalian cells and is conserved in *C*. *elegans* [9, 14]. Accordingly, *pas-5* RNAi treatment increased the total level of proteasome α-subunits compared to the control treatment, when whole animal lysates were analyzed by Western blotting with the anti-20S antibody (Fig 2A and 2B). We next wanted to investigate whether the *pas-5* knockdown would affect proteasome 20S α-subunit expression in a tissue-specific manner. Interestingly, immunohistochemically stained sections of *C*. *elegans* revealed that widespread *pas-5* RNAi enhances proteasome immunoreactivity in some cell types, such as intestinal and body-wall muscle cells, whilst a reduction in expression was detected in oocytes, germ cells and in embryos compared to the control treatment (Fig 2C and 2D). A similar trend in proteasome expression was also detected upon *pas-6* RNAi treatment (S3A and S3B Fig). The *pas-5* and *pas-6* RNAi mediated induction was most prominent in the intestine, particularly in the cytoplasm in response to both *pas-5* and *pas-6* RNAi (Fig 2D and S3B Fig). The *pas-5* RNAi mediated enhancement in proteasome expression was abolished upon simultaneous knockdown of *pas-5* and *skn-1*, showing a SKN-1 dependency in the bounce-back response (Fig 2E and 2F). To test for variation in RNAi tissue efficiency, we investigated whether downregulation of *gfp* is reflected in the GFP immunoreactivity in different tissues. The *gfp* RNAi experiment showed a similar trend of decreased expression of GFP in both intestinal and body-wall muscle cells, as detected by immunohistochemical and live fluorescence analyses (S4A and S4B Fig). Taken together, our results reveal that proteasome expression varies in different tissues and cell compartments in response to widespread RNAi targeting distinct proteasome subunits.

### Long-lived *daf-2* mutants show similar proteasome tissue expression levels as aged-matched wild-type animals

**Fig 3:**
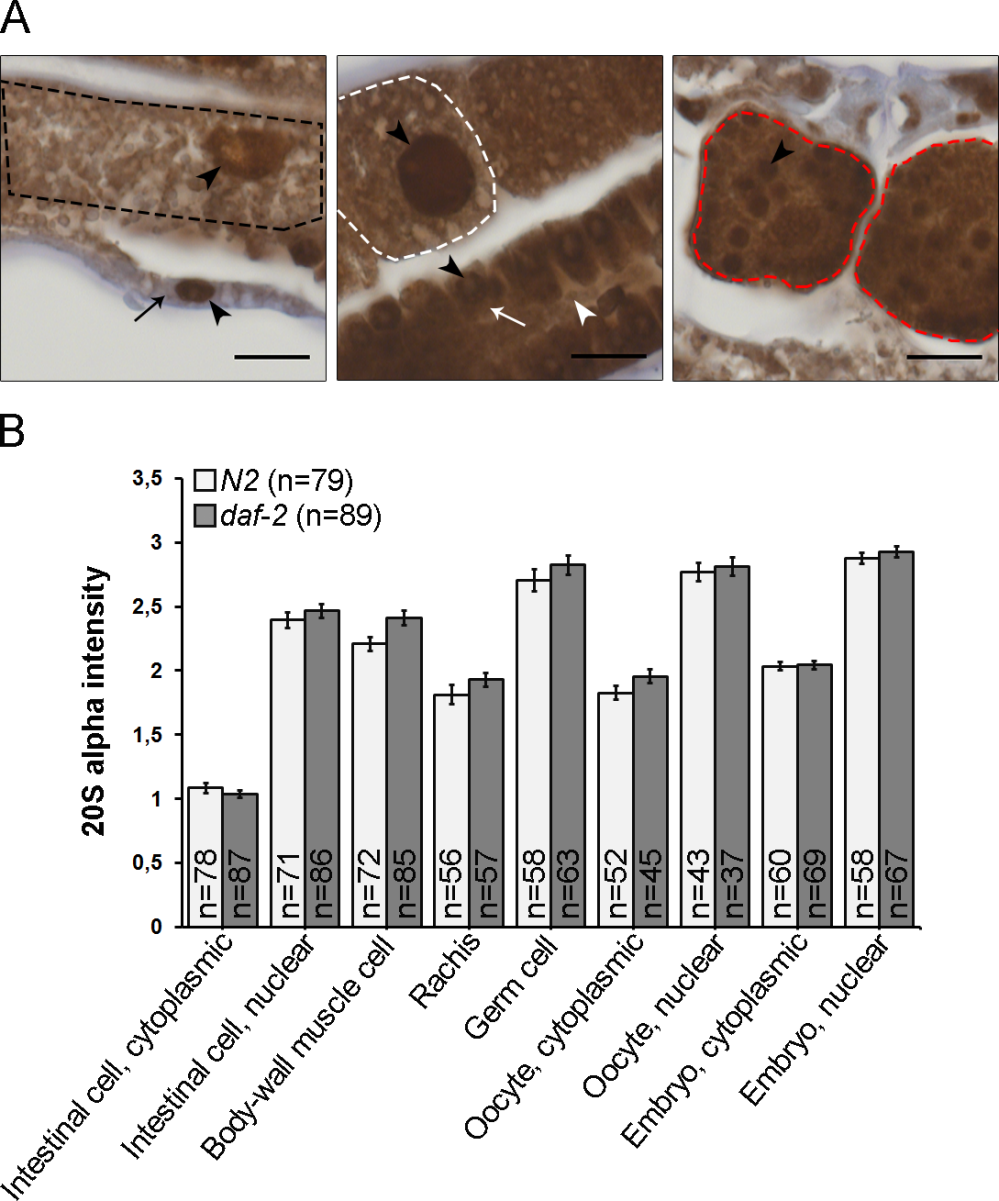
Proteasome tissue expression is similar in long-lived *daf-2(e1370)* mutants and wild-type animals. (A) Large magnification images of a long-lived *daf-2(e1370)* mutant showing proteasome immunoreactivity in an intestinal cell (left panel, outlined with a black dash line), body-wall muscle cell (left panel, indicated by a black arrow), germ cells (middle panel, indicated by a white arrow), rachis (middle panel, indicated by a white arrowhead), oocytes (middle panel, outlined with a white dash line), and embryos (right panel, outlined with red dash lines). Black arrowhead points to nucleus. Scale bars: 10 μm. (B) Quantification of immunoreactivity. Graph shows the mean staining intensity of three independent experiments (n = number of animals). Wild-type staining intensity data are the same as used in Fig 1D. Error bars ± SEM. No clear differences (≥10%) were detected in immunoreactivity.

It has previously been reported by various groups that there is either no difference, a reduction or an increase in proteasome protein levels in lysates of long-lived *daf-2* animals compared to lysates of wild-type animals [7, 24–26]. We have demonstrated that *daf-2(e1370)* animals exhibit increased *in vivo* proteasome activity in intestinal and body-wall muscle cells compared to wild-type animals, but without any detectable difference in proteasome amount in whole animal lysates analyzed by Western blotting [7].Therefore, there is a need to more accurately examine proteasome protein expression levels in tissues of *daf-2(e1370)* mutants. Immunohistochemically stained sections of 1-day old adult *daf-2(e1370)* mutants revealed a similar proteasome immunoreactivity pattern as in aged-matched wild-type animals, showing proteasome expression in intestinal cells, body-wall muscle cells, and in the gonad, including germ cells, oocytes, and embryos (Figs 3A and 1C). As in wild-type sections, the proteasome immunoreactivity in adult *daf-2(e1370)* mutants was stronger in the nucleus and/or perinuclear space, compared to the cytoplasm (Figs 3A and 1C). No clear tissue- or cell-compartment specific differences were detected in proteasome immunoreactivity between *daf-2(e1370)* mutants and wild-type sections (Fig 3B). Taken together, we show that proteasome expression is similar at the tissue level between young adult *daf-2(e1370)* and wild-type animals.

## Discussion

We present here the first report on proteasome tissue expression in *C*. *elegans* and demonstrate that the response to a stress condition can result in tissue-specific variations in proteasome levels. Our study demonstrates the need for a complementary approach with preserved tissue resolution to the commonly used whole animal lysate-based methods for studies on abundance of proteasome as well as of other proteins in metazoans.

We performed immunohistochemistry on thin *C*. *elegans* longitudinal sections with an antibody against six out of the seven proteasomal α-subunits, to reflect the endogenous expression of the 20S proteasome. To our knowledge there is no corresponding antibody recognizing multiple β-subunits of the 20S proteasome. Detection of the 20S core particle was chosen as a proxy for proteasome expression, as the *in vivo* variations in the number and type of regulatory particles associated with the 20S proteasome [2, 3], may affect data interpretation. As it has been proposed that “free” α-type subunits may exist in late passage human embryonic fibroblasts [27], we tested whether we could detect free α-subunits in worm lysates. Importantly, the anti-20S α-subunit antibody detected α-subunits only as parts of the 20S and/or 26S proteasome complexes, showing that positive immunoreaction corresponds to endogenous proteasome expression in *C*. *elegans* sections. Our analyses of GFP expressing animals confirm that the intensity and pattern of immunoreactivity represent the detected *in vivo* GFP fluorescence, demonstrating the suitability of immunohistochemistry for detection of protein expression in *C*. *elegans* sections. We reveal that proteasome is expressed in most tissues and cell types in an adult wild-type *C*. *elegans*, which is in agreement with the ubiquitous proteasome expression reported for female *Drosophila melanogaster* [28]. As previously shown in mammalian cells [29], we demonstrate that the proteasome is localized both in the cytoplasm and nucleus in all observed cell-types in *C*. *elegans*. Importantly we show that the proteasome expression pattern varies between cell types, in particularly the ratio between nuclear and cytoplasmic staining.

It is well-known that eukaryotic cells try to compensate for proteasome impairment by producing more proteasomes. This coordinated up-regulation of proteasome subunits upon impaired proteasomal activity was first reported in mammalian cells and is dependent on the transcription factor Nrf1/2 [9, 10, 12, 13]. We have previously demonstrated that this so called “bounce-back” response also functions in *C*. *elegans* and is largely dependent upon the Nrf orthologue SKN-1 [14]. Specifically, we showed that knockdown of *pas-5* by RNAi increases the *in vivo* expression of transcriptional and translational reporters of proteasomal genes predominantly in the intestine, but also in muscle cells [14]. In agreement, we here reveal that targeted knockdown of proteasome subunit *pas-5* increases proteasome expression predominantly in intestinal cells, but also in body-wall muscle cells. This strong *pas-5* RNAi mediated enhancement in proteasome expression in the intestine is dependent on SKN-1, as also reported previously [14]. Interestingly, we also observed a reduction in immunoreactivity in embryos and some cells related to reproduction. It is worth noting that *pas-5* or *pas-6* RNAi experiments were started at L3 larval stage to facilitate development into adulthood, as genes encoding the 20S core proteasome are essential for *C*. *elegans* development and RNAi experiments started at L1 larval stage induce developmental arrest (data not shown and [30]). Thus, the downregulation of *pas-5* or *pas-6* does not seem to be rate limiting for assembly of new proteasomes. Taken together, our data establish for the first time that there are tissue- and cell-type specific variations in endogenous proteasome expression during the “bounce back” response in *C*. *elegans*.

Insulin/IGF-1 signaling (IIS) pathway is a well-characterized evolutionarily conserved pathway involved in maintaining cellular protein homeostasis and several quantitative proteomic studies have been performed in long-lived *daf-2* IIS mutants. Dong *et al*. [24] detected no differential expression of 20S proteasome subunits between *daf-2(e1370)* mutants and N2 animals at day 1 of adulthood, while a study by Stout *et al*. [25] showed decreased expression of most proteasome core subunits in *daf-2(1370)* mutants compared to N2 animals at first day of adulthood. A recent quantitative proteomic study revealed an aging-induced accumulation, at time points between day 6 and day 22 of adulthood, of most of 20S and 19S subunits in N2 animals [26]. This accumulation was enhanced in *daf-2(e1370)* mutants [26]. We have previously shown by Western blotting that there is no difference in the total amount of proteasome alpha-subunits in whole animal lysates from young *daf-2(e1370)* adults and N2 animals [7]. Here, our immunohistochemistry data further confirm that there are no differences in proteasome expression at the tissue level between *daf-2(e1370)* and wild-type animals at day 1 of adulthood. This identical proteasome tissue expression is intriguing, taking into account that we have previously shown that *daf-2(e1370)* mutants display increased *in vivo* UPS activity for example in intestinal cells compared to wild-type animals [7]. This could at least partially be explained by our previous finding that the proteasome-associated deubiquitinating enzyme UBH-4 regulates proteasome activity and that *ubh-4* expression is negatively regulated in the intestine by the decreased IIS of the *daf-2(e1370)* mutants. Our results are in support of recent studies showing that regulation of protein turnover in *daf-2* mutants is a complex process [7, 31–33].

Immunohistochemistry has both advantages and limitations. The intensity of the immunoreactivity might be affected by cell-specific features, such as size and morphology. This semi-quantitative method, however, is suitable for comparing intensity variations in the same cell type(s) or tissues upon different treatments or in different strains. It is however possible that small changes in protein expression in the cell may not be within the detection range of this semi-quantitative antibody-based method. Compared to whole animal immunofluorescence experiments, immunohistochemistry on *C*. *elegans* sections facilitates efficient antibody tissue accessibility.

Taken together, our results reveal that a stress condition may elicit tissue-specific proteasome expression responses in *C*. *elegans*. Moreover, our data highlight the importance of implementing a method that captures changes in protein expression occurring at cell or tissue resolution level in a multicellular organism.

## Supporting information

S1 Fig Testing the specificity of the antibody against proteasome 20S α-subunits. (A) Lysates of wild-type animals separated on SDS-PAGE prior to immunoblotting with the anti-proteasome 20S α-subunits antibody (upper panel). Lower panel shows α-tubulin expression. (B) Lysates of wild-type animals separated under native conditions prior to immunoblotting with the anti-proteasome 20S α-subunits antibody. RP_2_-CP and RP-CP correspond to 26S with two or one 19S regulatory particle, respectively. CP corresponds to 20S core particle. (C) Lysates of wild-type animals separated on a native gel followed by in-gel proteasome activity assay with fluorogenic suc-LLVY-AMC substrate. RP_2_-CP and RP-CP correspond to 26S with two or one 19S regulatory particle, respectively. CP corresponds to 20S core particle. (D) Formalin-fixed, paraffin-embedded wild-type adult (4-day old) *C*. *elegans* sections showing immunoreactivity with anti-20S α-antibody (left panel), with omitted primary antibody (middle panel), and after pre-absorption with purified human 20S proteasome (right panel). Scale bars: 100 μm.
(TIF)

S2 Fig Validating suitability of enzymatic, peroxidase-based immunohistochemistry for inter-tissue and sub-cellular protein expression comparison. (A) Image presenting GFP immunoreactivity in a formalin-fixed, paraffin-embedded sections of an adult animal expressing GFP as a fusion protein under the control of the ubiquitous *sur-5* promoter. Intestinal cells are outlined with white dash lines and a body-wall muscle cell is indicated by a white arrow. Scale bar: 50 μm. Inserted an enlarged image of the indicated area with arrowheads pointing to nuclei of intestinal and muscle cells. (B) Representative GFP fluorescence micrograph (left panel) and an overlay with the bright-field micrograph (right panel). Nuclei of body-wall muscle cells and intestinal cells are indicated with a white arrow and with an arrowhead, respectively. Scale bars: 50 μm. Inserted an enlarged image of the indicated area.
(TIF)

S3 Fig Knockdown of *pas-6* similarly affects proteasome expression in the intestine as *pas-5* RNAi. (A) Images of an adult wild-type animal fed with control (left panel) or *pas-6* RNAi bacteria (right panel) presenting proteasome immunoreactivity in intestinal cells (outlined with black dash lines). Black arrowhead points to nucleus Scale bars: 10 μm. (B) Quantification of immunoreactivity. Graph shows the mean staining intensity of two independent experiments (n = number of animals). Error bars ± SEM. ***p < 0,001.
(TIF)

S4 Fig Testing of RNAi efficiency. (A) Images presenting GFP immunoreactivity in a control (left panel) or *gfp* RNAi (right panel) treated GFP-expressing animals. Intestinal cells are outlined with white dash lines and body-wall muscle cells are indicated by white arrows. White arrowhead points to nucleus. Scale bars: 10 μm. (B) Representative GFP fluorescence micrographs of animals treated with control RNAi (upper left panel) or *gfp* RNAi (upper right panel). Lower panels show overlay of bright-field micrograph and fluorescence micrograph. Body-wall muscle cells are indicated with white arrows and intestinal cells with white arrowheads. Scale bars: 10 μm. Please note that the GFP fluorescence in the intestine is overexposed to enable visualization of body-wall muscle cell GFP expression in the same image.
(TIF)
