# Supplementary figures and images for: Immunohistochemical analysis reveals variations in proteasome tissue expression in C. elegans
Source: PLoS One. 2017 Aug 17;12(8):e0183403. doi: 10.1371/journal.pone.0183403 (PMC5560697; doi:10.1371/journal.pone.0183403)

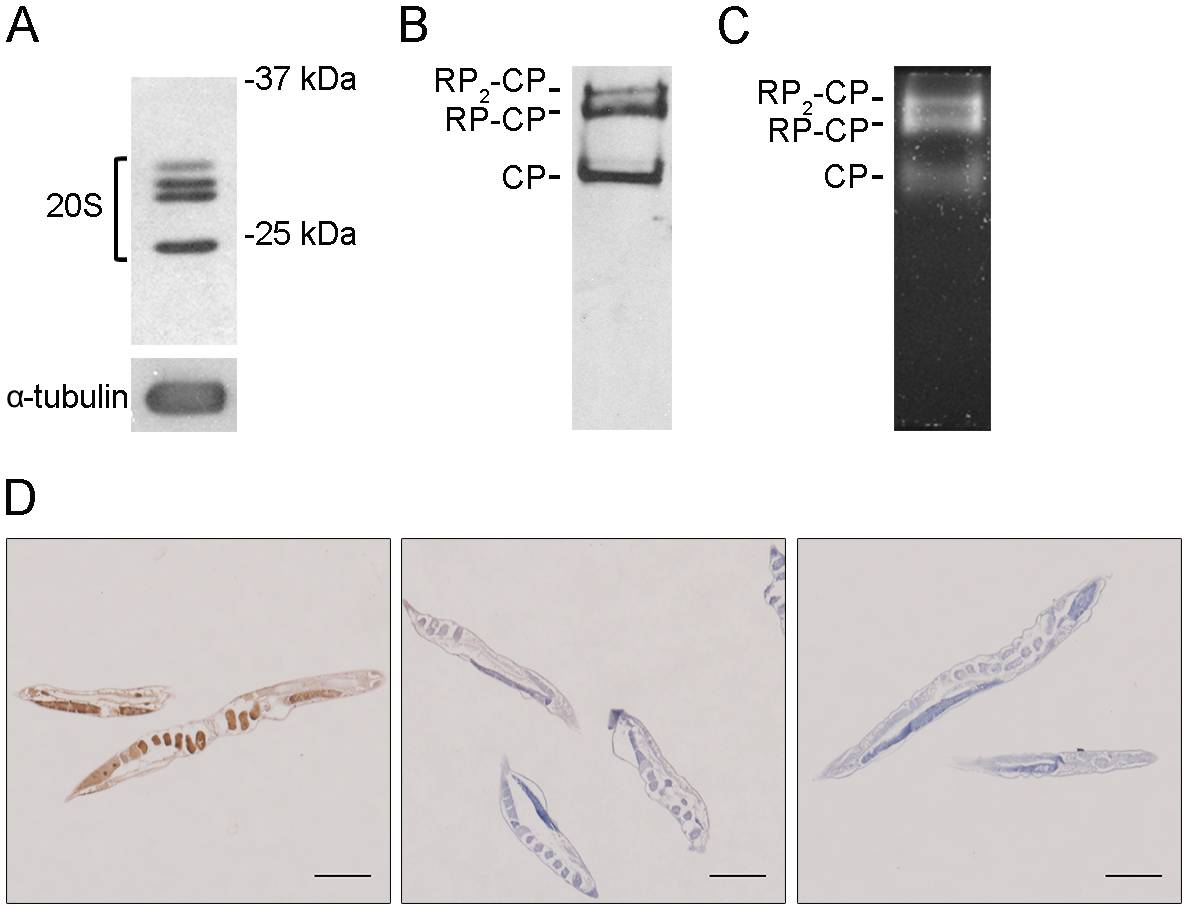

Supplement: S1 Fig — (A) Lysates of wild-type animals separated on SDS-PAGE prior to immunoblotting with the anti-proteasome 20S α-subunits antibody (upper panel). Lower panel shows α-tubulin expression. (B) Lysates of wild-type animals separated under native conditions prior to immunoblotting with the anti-proteasome 20S α-subunits antibody. RP2-CP and RP-CP correspond to 26S with two or one 19S regulatory particle, respectively. CP corresponds to 20S core particle. (C) Lysates of wild-type animals separated on a native gel followed by in-gel proteasome activity assay with fluorogenic suc-LLVY-AMC substrate. RP2-CP and RP-CP correspond to 26S with two or one 19S regulatory particle, respectively. CP corresponds to 20S core particle. (D) Formalin-fixed, paraffin-embedded wild-type adult (4-day old) C. elegans sections showing immunoreactivity with anti-20S α-antibody (left panel), with omitted primary antibody (middle panel), and after pre-absorption with purified human 20S proteasome (right panel). Scale bars: 100 μm. (TIF) [file pone.0183403.s001.tif]

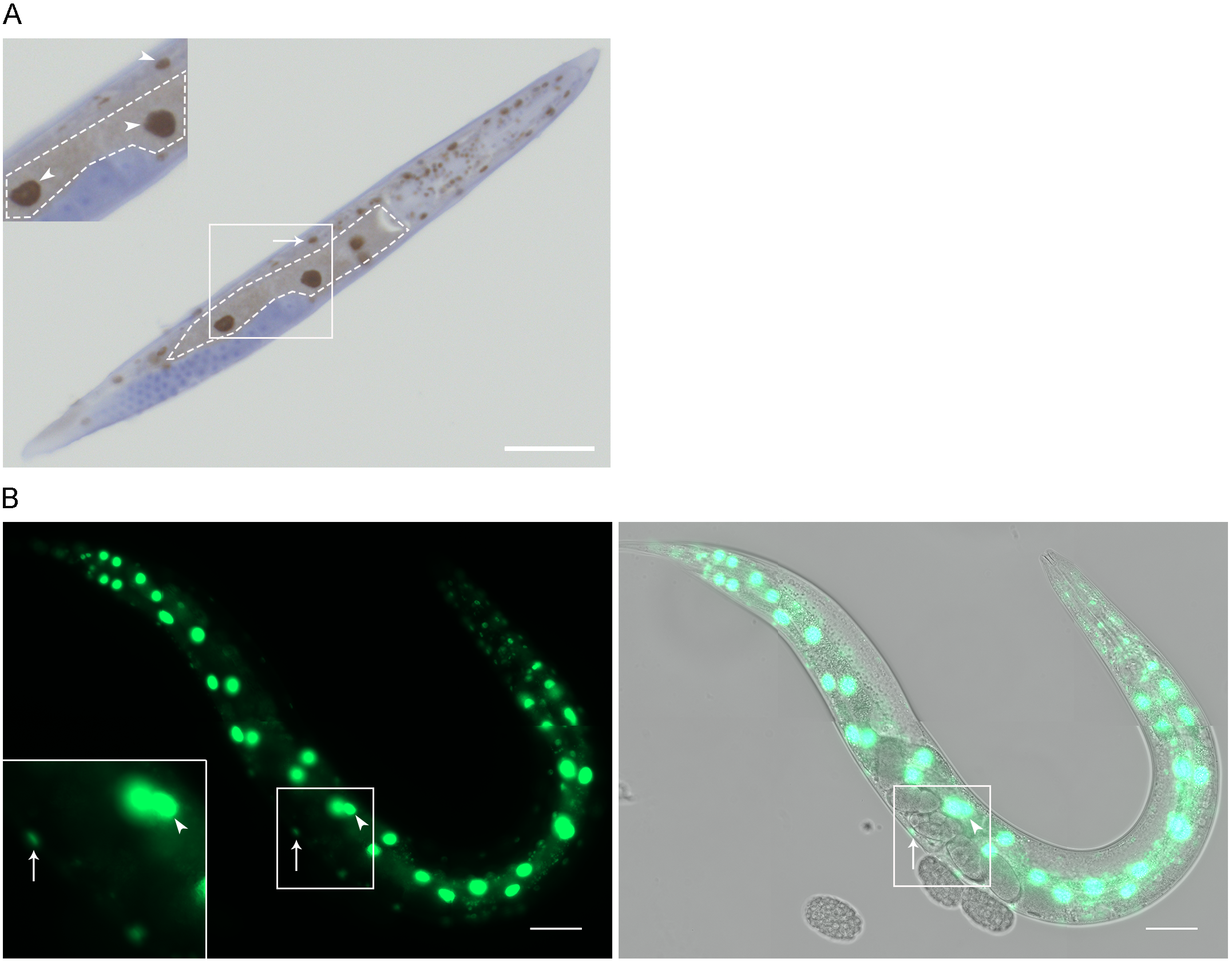

Supplement: S2 Fig — (A) Image presenting GFP immunoreactivity in a formalin-fixed, paraffin-embedded sections of an adult animal expressing GFP as a fusion protein under the control of the ubiquitous sur-5 promoter. Intestinal cells are outlined with white dash lines and a body-wall muscle cell is indicated by a white arrow. Scale bar: 50 μm. Inserted an enlarged image of the indicated area with arrowheads pointing to nuclei of intestinal and muscle cells. (B) Representative GFP fluorescence micrograph (left panel) and an overlay with the bright-field micrograph (right panel). Nuclei of body-wall muscle cells and intestinal cells are indicated with a white arrow and with an arrowhead, respectively. Scale bars: 50 μm. Inserted an enlarged image of the indicated area. (TIF) [file pone.0183403.s002.tif]

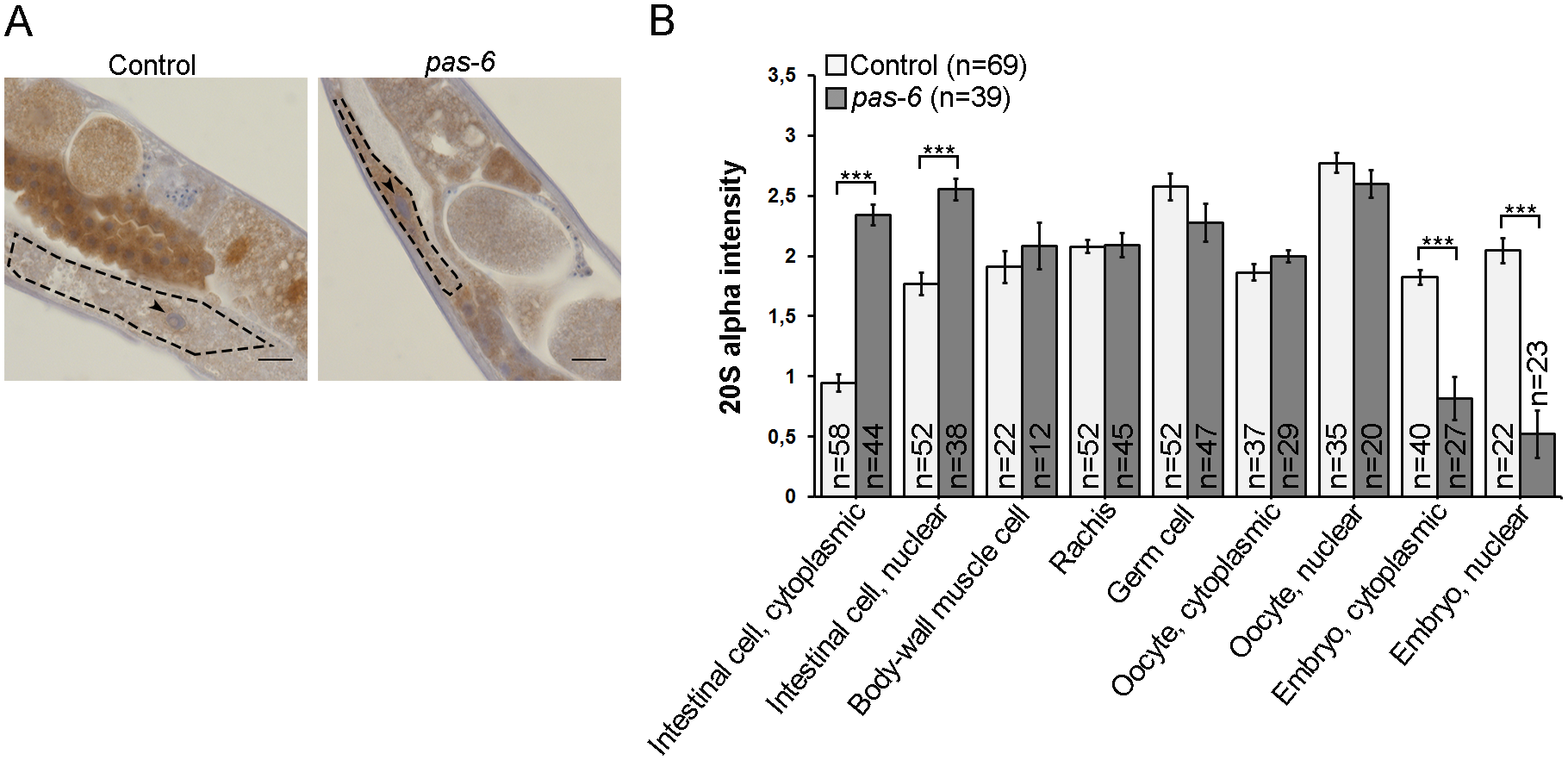

Supplement: S3 Fig — (A) Images of an adult wild-type animal fed with control (left panel) or pas-6 RNAi bacteria (right panel) presenting proteasome immunoreactivity in intestinal cells (outlined with black dash lines). Black arrowhead points to nucleus Scale bars: 10 μm. (B) Quantification of immunoreactivity. Graph shows the mean staining intensity of two independent experiments (n = number of animals). Error bars ± SEM. ***p < 0,001. (TIF) [file pone.0183403.s003.tif]

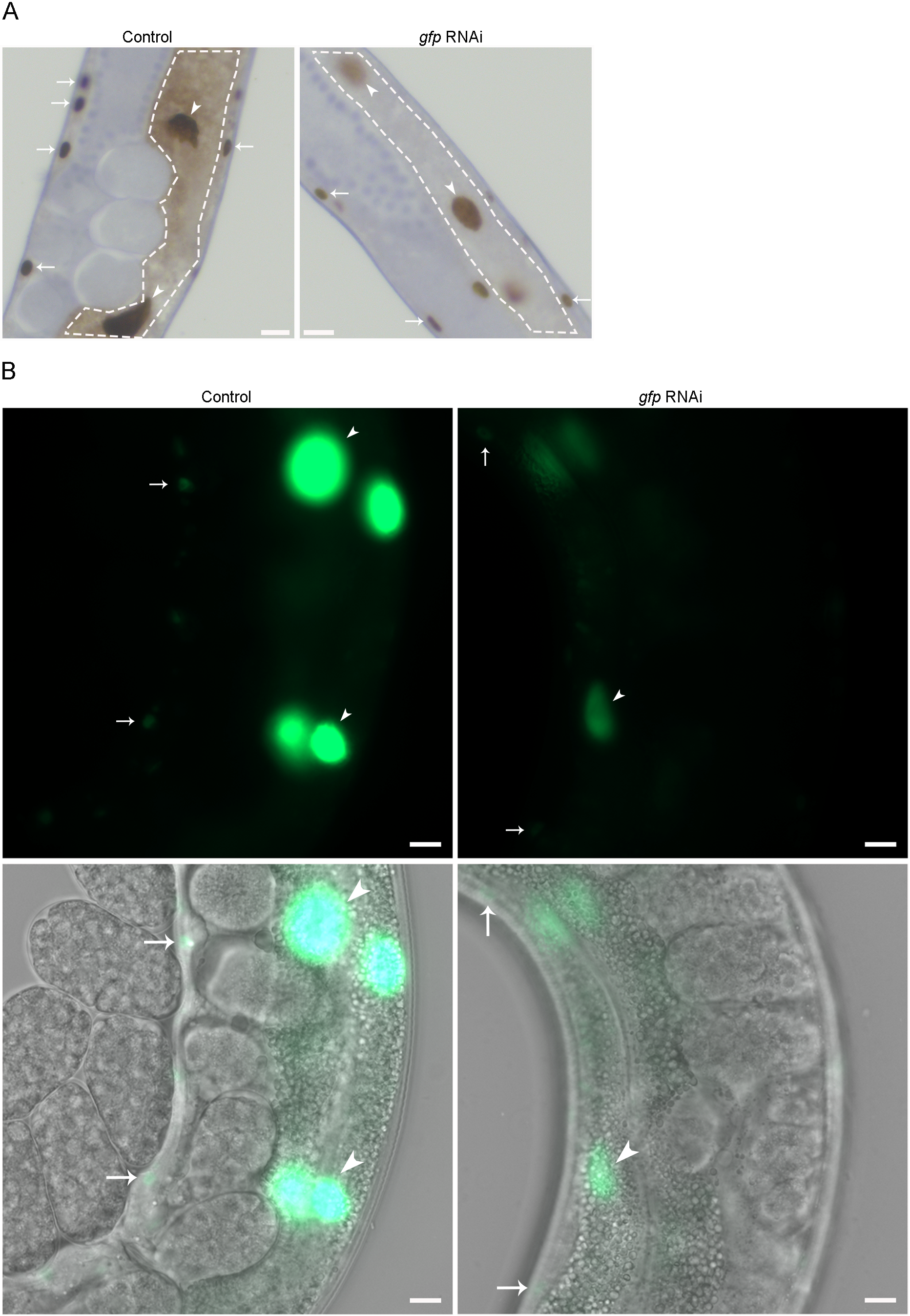

Supplement: S4 Fig — (A) Images presenting GFP immunoreactivity in a control (left panel) or gfp RNAi (right panel) treated GFP-expressing animals. Intestinal cells are outlined with white dash lines and body-wall muscle cells are indicated by white arrows. White arrowhead points to nucleus. Scale bars: 10 μm. (B) Representative GFP fluorescence micrographs of animals treated with control RNAi (upper left panel) or gfp RNAi (upper right panel). Lower panels show overlay of bright-field micrograph and fluorescence micrograph. Body-wall muscle cells are indicated with white arrows and intestinal cells with white arrowheads. Scale bars: 10 μm. Please note that the GFP fluorescence in the intestine is overexposed to enable visualization of body-wall muscle cell GFP expression in the same image. (TIF) [file pone.0183403.s004.tif]
